# West Nile virus spread in Italy, summer 2025: a climate change hallmark?

**DOI:** 10.3389/fpubh.2025.1722110

**Published:** 2025-12-08

**Authors:** Rita Cuciniello, Flavia Pennisi, Anna Carole D'Amelio, Carlo Signorelli, Giovanni Rezza

**Affiliations:** 1 School of Medicine, University Vita-Salute San Raffaele, Milan, Italy; 2 PhD National Programme in One Health Approaches to Infectious Diseases and Life Science Research, Department of Public Health, Experimental and Forensic Medicine, University of Pavia, Pavia, Italy

**Keywords:** vector borne disease, arboviruses, West Nile virus, One Health, climate change

West Nile virus (WNV), a flavivirus transmitted by *Culex* spp. mosquitoes, has caused several outbreaks in the Mediterranean area and central Europe since the 1950s, with recrudescence every hot season (1). Birds are amplifying hosts and may play an important role in dispersing the virus both through local movements of erratic resident birds and long-range travel of migratory birds (2).

Several factors may increase the risk of WNV outbreaks, including climate change, which consists of a warming atmosphere characterized by heat waves and changing weather conditions (i.e., increased precipitation) (3, 4). Intensity of transmission is determined by the abundance of mosquitoes and prevalence of infection (5).

We went through the data of surveillance reports on WNV in Italy (6), to evaluate changes in the distribution of human cases over the years, and to discuss factors contributing to variations in the spread of the infection/disease. In particular, we tried to interpret changes in the pattern of human cases observed in the year 2025.

In Italy, after an outbreak among horses in 1998, the virus reappeared in 2008 (7). Since then, there has been a seasonal resurgence of cases, especially in the Po Valley (Pianura Padana), a wide plain area sited in the north of the Country. Typically, the number of cases begins to increase in July, with peaks in August and a decline in September; however, annual fluctuations have been observed, with the highest incidence rates reported in 2018 (8) and 2022 (9).

This year, the number of cases was already high in July, with a modest increase and a peak in early August (680 WNV cases, of whom 321 neuroinvasive and 48 deaths until September 25, 2025).

Although the incidence rate of neuroinvasive cases was comparable to that reported in previous years, the geographic area affected by WNV cases was notably broader, involving 17 of the 21 Regions and Autonomous Provinces and encompassing a total of 74 out of 110 provinces, with the number of neuroinvasive cases ranging from 1 to 60 (6).

The early emergence of cases may be at least in part explained by the high temperatures and heat waves registered in June, while the reasons for the large extension of the national territory affected by WNV remain undefined. In fact, the dynamics of WNV are extremely complex. While most cases were concentrated in few provinces of two Regions (the province of Latina in the Lazio Region, and those of Caserta and Naples in Campania), the other cases were scattered in the traditionally affected Regions in Po Valley, in western Sardinia, but also in other areas of the Country. Thus, changes in the pattern of WNV cases consisted mainly in a broader geographical distribution of human cases, which was confirmed also by the detection of the virus in vectors and several animal species (10).

There are several questions that need to be answered: which are the factors determining the spread of the virus outside established “endemic” areas? What factors may explain a high number of cases in a certain area beyond the density of mosquitoes, and what is the role played by the spread of the virus among resident birds, bird migration patterns, or other environmental conditions? Are sporadic cases dispersed through the national territory outside high incidence foci, the consequence of bird erratic movements through geographical sites contiguous to those heavily affected or does the virus jump from one place to another through long-range movement of migratory birds? At last, to what extent does the age distribution of the population affect the infected-to-ill cases?

Early detection of viral circulation in mosquitoes and both resident and migratory birds is essential to allow public-health authorities to anticipate and optimize vector-control measures and better target preventive actions. To this end, strengthening integrated surveillance systems, combining entomological, avian, and human data, remains a cornerstone for intervention strategies in settings where seasonal resurgence is expected (11–13).

In conclusion, WNV poses important challenges to temperate climate countries because of annual recrudescence. Since vaccines have not gone beyond phase 2 trials (4), preparedness and response are essential to reduce the spread of the virus during the hot season and its clinical impact.
